# Characteristics of women infected with human papillomavirus in a tertiary hospital in Beijing China, 2014–2018

**DOI:** 10.1186/s12879-019-4313-8

**Published:** 2019-07-29

**Authors:** Liang Ma, Jieping Lei, Li Ma, Xiao Cong, Na Wang, Hui Yang, Qian Liu, Yang Yu, Yongtong Cao

**Affiliations:** 1 Clinical Laboratory, China-Japan Friendship Hospital, Beijing, People’s Republic of China; 2 Data and Project Management Unit, Institute of Clinical Medical Sciences, China-Japan Friendship Hospital, Beijing, China; 3 Institute of Respiratory Medicine, Chinese Academy of Medical Sciences, Beijing, China; 4 National Clinical Research Center for Respiratory Diseases, Beijing, China; 5 Department of Gynaecology & Obstetrics, China-Japan Friendship Hospital, Beijing, China; 6 Department of Blood Transfusion, China-Japan Friendship Hospital, Beijing, China

**Keywords:** Human papillomavirus, Genotype, ThinPrep cytological test

## Abstract

**Background:**

Human papillomavirus (HPV) infection is the main cause of precancerous lesions and cervical cancer in women. In order to determine the epidemiological characteristics as well as the relationship between the HPV genotype and cytology test results among women in Beijing, China, we retrospectively collected and analyzed the data from a tertiary hospital in Beijing, China.

**Methods:**

A total of 21,239 women visited the China–Japan Friendship Hospital between 2014 and 2018 and their cervical exfoliations were collected. Thirteen HPV subtypes (16, 18, 31, 33, 35, 39, 45, 51, 52, 56, 58, 59 and 68) were examined and ThinPrep cytological test (TCT) was performed.

**Results:**

Among all cases, 4473 (21.06%) women were infected with HPV. HPV52 (4.64%), HPV16 (4.44%) and HPV58 (4.28%) had higher prevalence. Single-type infection (77.26%) was more common than multiple-type infection (22.74%). Single-type infection was more frequently seen in women aged 50–60 years (17.17%), and multiple-type infection was more common in those aged < 30 years (7.88%). Significant differences in secular trends from 2014 to 2018 were observed for subtypes HPV39, 51, 52 and 58. HPV positive rates of women aged < 30 and 30–40 years changed significantly along with the time period, and the TCT positive rates of women aged 30–40, 40–50, 50–60 and >  60 years also showed significant differences from 2014 to 2018. In addition, 1746 (8.22%) women were TCT positive, of whom, 858 (4.04%), 561 (2.64%) and 327 (1.54%) had atypical squamous cells (ASCs), low-grade squamous intraepithelial lesions (LSILs) and high-grade squamous intraepithelial lesions (HSILs), respectively. Among four types of cytological lesions, the HPV infection rates were 16.76, 66.08, 63.99 and 85.32% in those negative for intraepithelial lesions or malignancy (NILM), ASC, LSIL and HSIL, respectively.

**Conclusions:**

HPV52, 16 and 58 are the most common infection subtypes in this study and among four types of cytological lesions, HSILs has the highest HPV prevalence. Significant differences in secular trends are observed for different subtypes in recent 5 years. The results on HPV genotype-specific prevalence should be considered when the HPV vaccine program is implemented in Beijing area.

## Background

Cervical cancer is the fourth most commonly encountered malignancy among women worldwide [1]. It is estimated that 85% of the 528,000 new cases and 87% of the 266,000 deaths from cervical cancer occurred in developing countries in 2012 [2]. In China, cervical cancer ranks seventh and ninth in cancer prevalence and mortality in women, respectively [3]. Persistent human papillomavirus (HPV) infection, especially high-risk HPV (HR-HPV) infection, is the main cause of precancerous lesions and cervical cancer [4, 5]. HPV is a small double-stranded, circular DNA virus that belongs to the family Papillomavirus [6]. There are 200 HPV genotypes that have been identified until now, and HR-HPVs are closely associated with cervical and anogenital tract cancers [7, 8].

HPV prevalence and genotype distribution differ geographically, and also between rural and urban women [8–11]. Epidemiological characteristics of HPV infection may also alter with population shift and habit changes. Understanding the characteristics is important and necessary to develop the HPV-based cervical cancer screening program and vaccine-based HPV prevention strategies.

Early-stage detection or diagnosis plays an important role in the prevention and treatment of cervical cancer. During recent years, with the development of cervical cancer screening programs, as well as the application of ThinPrep cytological test (TCT), the detection rate of cervical lesions has improved. HPV testing has been shown to be a reasonable option and widely used in the screening of cervical precancerous diseases [12]. HPV testing alone has also been developed as the primary screening modality in several countries [13]. However, HPV testing is too sensitive to reflect cervical lesions, and HPV can be eliminated by the immune system before causing cervical lesions. Therefore, HPV testing shows high negative predictive value (NPV) and poor positive predictive value (PPV), especially in women aged < 30 years with high HPV infection rate [14, 15]. Furthermore, HPV testing may fail to detect cervical diseases that could be diagnosed by other methods. For instance, TCT could improve the screening efficiency of cervical cytology and provide a better medium for cervical cells [16]. The experience in the US and Europe showed that cytological screening on its own or combined with HPV testing decreased the incidence rate of cervical cancer [17]. The American Cancer Society, the American Society for Colposcopy and Cervical Pathology, and the American Society of Clinical Pathology have suggested that the combination of cytology and HPV testing significantly increase the sensitivity of cervical cancer screening [18]. Several large studies have been conducted to compare the efficacy of cytology and HPV testing in detecting precancerous lesions and cervical cancer [19–21]. In addition, a few studies have focused on the relationship between the HPV genotype and cytology test results in China.

In this study, all participants underwent cytology testing and HPV detection on the same visit. The aim of the study was to determine the epidemiological characteristics and secular trends of HPV infection subtypes in the Beijing area. We also analyzed the relationship between HPV genotype and cytology test results. We retrospectively analyzed the data from a tertiary hospital in Beijing. The results will contribute to the data on HPV genotype-specific prevalence in the Beijing area in order to encourage implementation of an HPV vaccine program.

## Methods

### Patient recruitment and sample collection

The China–Japan Friendship Hospital is a tertiary hospital that provides medical care in the Beijing area. We retrospectively collected the data of 21,239 women who visited the hospital and received TCT for HPV detection between 2014 and 2018. The patients’ confidentiality was protected by hiding their identities. Written informed consent was obtained from all the patients prior to their enrollment in the study. The study design adhered to the principles of the Declaration of Helsinki and was approved by the ethics committee of China-Japan Friendship Hospital.

### Cytology testing

The samples were strictly collected during the nonmenstrual period. Cervical liquid-based cytology tests were performed by experienced cytology experts at the Department of Gynaecology & Obstetrics. The terminology for liquid-based cytology was defined according to the Bethesda System and included negative for intraepithelial lesions or malignancy (NILM), atypical squamous cells of undetermined significance (ASC-US), low-grade squamous intraepithelial lesion (LSIL), and high grade squamous intraepithelial lesion (HSIL) [22].

### HPV DNA testing

Exfoliated cervical cells were collected using a specialized cervical brush. Then, the cervical samples were stored at 4 °C in standard medium provided by Liferiver (Shanghai, China). DNA isolation and purification were performed. A Chinese State Food and Drug Administration-approved HPV genotyping assay (Liferiver) was used to detect HPV genotypes by real-time polymerase chain reaction. This test individually detected 13 high-risk HPV subtypes, including HPV16, 18, 31, 33, 35, 39, 45, 51, 52, 56, 58, 59 and 68. Amplification was performed using a real-time polymerase chain reaction (PCR) detector, and the profile consisted of denaturation at 94 °C for 2 min following 40 cycles of denaturation at 93 °C for 10 s, annealing and elongation at 62 °C for 31 s. Single point fluorescence was detected at 62 °C. Internal quality control and External quality assessment were taken in the experiment and the results met the requirements.

### Statistical analysis

Data were presented as mean ± standard deviation or frequency and percentage for numerical or discrete variables, respectively. The χ^2^ test was used to determine whether significant difference existed between groups for discrete variables. The Cochran–Armitage trend test was used to determine the statistical significance of changes over years. All analyses were performed using SAS 9.4 software (Cary, NC, USA).

## Results

### Single and multiple type HPV infections in women of different ages

A total of 21,239 women (mean age 40.02 ± 11.38 years) visited the China–Japan Friendship Hospital and received cervical TCT and HPV tests between 2014 and 2018. Characteristics of HPV infection as well as HPV distribution among different age categories are shown in Table 1. Among all 21,239 cases, 4473 (21.06%) were infected with HPV, which was more frequently seen in 998 (998/4046, 24.67%) women aged < 30 years. Single-type HPV infection was more frequently seen in women aged 50–60 years (542/3157, 17.17%), and multiple-type HPV infection was more common in those aged < 30 years (319/4046, 7.88%). And apparently, in our data single-type HPV infection (3456/4473, 77.26%) was more common than multiple-type infection (1017/4473, 22.74%) (χ^2^ test *P* < 0.0001).

**Table 1:** Single and multiple type infections of HPV of different ages

Age (years)	N	HPV Positive, n (%^a^)	Single-type infection, n (%^a^)	Multiple-type infection, n (%^a^)
< 30	4046	998 (24.67)	679 (16.78)	319 (7.88)
30–40	7666	1567 (20.44)	1258 (16.41)	309 (4.03)
40–50	5014	949 (18.93)	773 (15.42)	176 (3.51)
50–60	3157	679 (21.51)	542 (17.17)	137 (4.34)
> 60	1356	280 (20.65)	204 (15.04)	76 (5.60)
Total	21,239	4473	3456	1017

*HPV* human papillomavirus

^a^ Represented HPV infection rates in different age categories

### Single and multiple type infection rates in women of different HPV subtypes

Among 13 HPV subtypes examined, HPV52, 16, and 58 had the higher prevalence in all included participants, with the infection rates of 4.64, 4.44 and 4.28%, respectively. The three HPV subtypes were also most commonly seen in women both with single and multiple HPV infections (Table 2).

**Table 2:** Single and multiple type infection rates of different HPV subtypes

HPV subtype	Positive, n (%^a^)	Single-type infection, n (%^a^)	Multiple-type infection, n (%^a^)
HPV16	942 (4.44)	630 (2.97)	312 (1.47)
HPV18	312 (1.47)	185 (0.87)	127 (0.60)
HPV31	264 (1.24)	158 (0.74)	106 (0.50)
HPV33	155 (0.73)	83 (0.39)	72 (0.34)
HPV35	205 (0.97)	87 (0.41)	118 (0.56)
HPV39	490 (2.31)	217 (1.02)	273 (1.29)
HPV45	86 (0.40)	43 (0.20)	43 (0.20)
HPV51	465 (2.19)	266 (1.25)	199 (0.94)
HPV52	986 (4.64)	655 (3.08)	331 (1.56)
HPV56	435 (2.05)	253 (1.19)	182 (0.86)
HPV58	909 (4.28)	595 (2.80)	314 (1.48)
HPV59	316 (1.49)	184 (0.87)	132 (0.62)
HPV68	258 (1.21)	99 (0.47)	159 (0.75)

*HPV* human papillomavirus

^a^ Represented HPV subtype infection rate which was calculated as the number of infected women by certain subtype of HPV was divided by the total number of participants in this study (*N* = 21,239)

### Secular trends of different HPV subtype infection rates, and HPV and TCT infection rates of different ages from 2014 to 2018

We also investigated the temporal trends of HPV subtype infections during the research period. HPV16, 52 and 58 constantly had the higher infection rates from 2014 to 2018. Significant differences in secular trends between 2014 and 2018 were seen for subtypes of HPV39, 51, 52 and 58 (all *P* < 0.05, Table 3).

**Table 3:** Secular trends of different HPV subtype infection rates from 2014 to 2018

HPV subtype	2014, n (%^a^)	2015, n (%^a^)	2016, n (%^a^)	2017, n (%^a^)	2018, n (%^a^)	*P* value for trend
HPV16	46 (3.91)	194 (4.23)	194 (3.94)	320 (5.01)	188 (4.52)	0.0644
HPV18	18 (1.53)	75 (1.63)	48 (0.97)	94 (1.47)	77 (1.85)	0.2684
HPV31	13 (1.11)	55 (1.20)	54 (1.10)	84 (1.32)	58 (1.39)	0.2386
HPV33	10 (0.85)	40 (0.87)	27 (0.55)	43 (0.67)	35 (0.84)	0.7999
HPV35	7 (0.60)	51 (1.11)	38 (0.77)	62 (0.97)	47 (1.13)	0.3404
HPV39	20 (1.70)	89 (1.94)	92 (1.87)	166 (2.60)	123 (2.96)	< 0.0001
HPV45	3 (0.26)	15 (0.33)	23 (0.47)	26 (0.41)	19 (0.46)	0.2899
HPV51	31 (2.64)	83 (1.81)	87 (1.76)	149 (2.33)	115 (2.76)	0.011
HPV52	46 (3.91)	182 (3.97)	174 (3.53)	356 (5.58)	228 (5.48)	< 0.0001
HPV56	31 (2.64)	93 (2.03)	94 (1.91)	138 (2.16)	79 (1.90)	0.4557
HPV58	54 (4.60)	179 (3.90)	178 (3.61)	306 (4.79)	192 (4.61)	0.0426
HPV59	12 (1.02)	71 (1.55)	62 (1.26)	100 (1.57)	71 (1.71)	0.1309
HPV68	21 (1.79)	63 (1.37)	58 (1.18)	55 (0.86)	61 (1.47)	0.2339
Total	1175	4588	4930	6384	4162	

*HPV* human papillomavirus

^a^ Represented HPV infection rates within each calendar year

We found that HPV positive rates in women aged < 30 years and 30–40 years changed significantly along with the time period (*P* value for trend = 0.0002 and < 0.0001 for women aged < 30 years and 30–40 years, respectively). Additionally, the TCT positive rates among women aged 30–40, 40–50, 50–60 and >  60 years also showed significant differences from 2014 to 2018 (Table 4).

**Table 4:** Secular trends of HPV and TCT infection rates of different ages from 2014 to 2018

Age (years)	2014, n (%^a^)	2015, n (%^a^)	2016, n (%^a^)	2017, n (%^a^)	2018, n (%^a^)	P value for trend
HPV positive						
< 30	47 (18.73)	183 (20.42)	208 (23.27)	344 (23.53)	216 (22.27)	0.0002
30–40	91 (36.25)	312 (34.82)	305 (34.12)	473 (32.35)	386 (39.79)	< 0.0001
40–50	48 (19.12)	201 (22.43)	175 (19.57)	339 (23.19)	186 (19.18)	0.0596
50–60	45 (17.93)	133 (14.84)	152 (17.00)	222 (15.18)	127 (13.09)	0.879
> 60	20 (7.97)	67 (7.48)	54 (6.04)	84 (5.75)	55 (5.67)	0.4306
Total	251	896	894	1462	970	–
TCT positive						
< 30	25 (18.12)	69 (16.67)	81 (19.52)	101 (19.13)	48 (19.12)	0.0541
30–40	48 (34.78)	131 (31.64)	147 (35.42)	170 (32.20)	104 (41.43)	0.016
40–50	32 (23.19)	118 (28.50)	96 (23.13)	144 (27.27)	49 (19.52)	< 0.0001
50–60	26 (18.84)	61 (14.73)	67 (16.14)	80 (15.15)	34 (13.55)	0.001
> 60	7 (5.07)	35 (8.45)	24 (5.78)	33 (6.25)	16 (6.37)	0.0385
Total	138	414	415	528	251	–

*HPV* human papillomavirus, *TCT* thinprep cytology test

^a^ Represented the percentage of HPV infection rates in different groups

### TCT positive infections in women of different ages

Characteristics of TCT test results as well as their distribution among different age categories are shown in Table 5. Among all participants, a number of 1746 (8.22%) women were TCT positive, of whom, 858 (4.04%), 561 (2.64%) and 327 (1.54%) had ASCs, LSILs and HSILs, respectively. The highest TCT positive rate was seen in women aged 40–50 years (8.76%), and the lowest rate was found in those women aged 30–40 years (7.83%). Additionally, among four cytological subtypes, HSIL detection increased significantly with age (*P* < 0.0001, Table 5). Moreover, among single-type and multiple-type HPV infections, the TCT positive rate was 24.77% (856/3456) and 34.32% (349/1017), respectively.

**Table 5:** TCT positive rates of different ages

Age (years)	N	NILM, n (%^a^)	ASC, n (%^a^)	LSIL, n (%^a^)	HSIL, n (%^a^)	TCT positive, n (%^a^)
< 30	4046	3722 (91.99)	181 (4.47)	123 (3.04)	20 (0.49)	324 (8.01)
30–40	7666	7066 (92.17)	300 (3.91)	198 (2.58)	102 (1.33)	600 (7.83)
40–50	5014	4575 (91.24)	212 (4.23)	131 (2.61)	96 (1.91)	439 (8.76)
50–60	3157	2889 (91.51)	115 (3.64)	80 (2.53)	73 (2.31)	268 (8.49)
> 60	1356	1241 (91.52)	50 (3.69)	29 (2.14)	36 (2.65)	115 (8.48)
Total	21,239	19,493	858	561	327	1746

*TCT* thinprep cytology test, *NILM* negative for intraepithelial lesions or malignancy, *ASC* atypical squamous cells, *LSIL* low grade squamous intraepithelial lesion, *HSIL* high grade squamous intraepithelial lesion

^a^ Represented TCT positive rates in different age categories

### Relationship between TCT test results and HPV subtype infections

Among four types of cytological lesions, the HPV infection rate was 16.76% (3268/19493), 66.08% (567/858), 63.99% (359/561), and 85.32% (279/327) for NILM, ASCs, LSILs and HSILs, respectively. HPV16, 52 and 58 were frequently determined in NILM, ASC and HSIL, and HPV16, 51 and 56 were commonly found in LSIL (Table 6).

**Table 6:** Different HPV subtype infection rates among NILM, ASC, LSIL, and HSIL, respectively

HPV subtype	NILM (%) *N* = 19,493	ASC (%) *N* = 858	LSIL (%) *N* = 561	HSIL (%) *N* = 327
HPV16	591 (3.03)	130 (15.15)	73 (13.01)	148 (45.26)
HPV18	234 (1.20)	46 (5.36)	16 (2.85)	16 (4.89)
HPV31	196 (1.01)	33 (3.85)	19 (3.39)	16 (4.89)
HPV33	103 (0.53)	26 (3.03)	14 (2.50)	12 (3.67)
HPV35	151 (0.77)	26 (3.03)	17 (3.03)	11 (3.36)
HPV39	368 (1.89)	68 (7.93)	41 (7.31)	13 (3.98)
HPV45	62 (0.32)	16 (1.86)	5 (0.89)	3 (0.92)
HPV51	296 (1.52)	79 (9.21)	76 (13.54)	14 (4.28)
HPV52	764 (3.92)	131 (15.27)	59 (10.52)	32 (9.79)
HPV56	272 (1.40)	57 (6.64)	98 (17.47)	8 (2.45)
HPV58	662 (3.40)	132 (15.38)	58 (10.34)	57 (17.43)
HPV59	253 (1.30)	39 (4.55)	18 (3.21)	6 (1.83)
HPV68	180 (0.92)	46 (5.36)	27 (4.81)	5 (1.53)

*HPV* human papillomavirus, *NILM* negative for intraepithelial lesions or malignancy, *ASC* atypical squamous cells, *LSIL* low grade squamous intraepithelial lesion, *HSIL* high grade squamous intraepithelial lesion

## Discussion

Persistent HR-HPV infection is the main cause of precancerous lesions and cervical cancer, which has been confirmed by epidemiological and biological data [23]. Active and effective detection methods for HPV are deemed important to control the development of cervical cancer. This study aimed at understanding the epidemiological characteristics of HPV infection subtypes and the relationship between the HPV testing and cytology test results in the Beijing area. Our study provided sufficient HPV genotyping data from a large Chinese female cohort in a single center. The results revealed that the total HPV infection rate was 21.06% in our study cohort, especially in women aged < 30 years. HPV16, 52 and 58 were the most commonly seen subtypes. The results also showed that the TCT positive rate was 8.22%, and among four types of cytological lesions, HSIL had the highest HPV infection rate.

Previous studies have reported that the frequency of HPV infection varies geographically [9, 24, 25]. The frequency of HPV infection also varies in China because of its large population and territory, and the frequency varies from 9.9 to 31.9% in different areas in China [26–28]. In our study, 21.06% of all 21,239 cases were infected with HPV. The frequency of HPV infection reached a peak in women aged< 30 years, gradually declined in the middle-aged groups, and then increased in women aged > 50 years. Our results are similar to those recently published by Jiang et al [28]. However, single-type HPV infection was more frequently seen in women aged 30–40 years, and multiple-type HPV infection was more common in those aged < 30 years. Women aged < 30 years are sexually active and may have more than one sexual partner, so the frequency of multiple-type HPV infection is higher than in other age groups. Most HPV infections can be cleared or suppressed within 1 or 2 years in 70–91% of cases [29]. Therefore, the higher HR-HPV prevalence among women aged > 50 years may be associated with reduced immunity. Our data also showed that single-type infection was more common than multiple-type infection. However, multiple-type infection is more dangerous than single-type infection. In this study, the TCT positive rate was higher in multiple-type than in single-type HPV infection, which is consistent with previous studies [30].

It is necessary to know the HPV genotype distribution for vaccine development. Among 13 HPV subtypes examined in our study, HPV52, 16, and 58 had the highest prevalence in overall, single-type and multiple-type infected participants. The results showed that the most common infection was with HPV52, which was detected in 4.64% of cases. HPV16 was found in 4.44% of cases and the frequency of HPV58 was 4.28%. These results were similar to previously reported data from other Chinese studies [28, 31, 32]. These studies demonstrated that both HPV52 and 58 were more common among the general population in China than in developed countries.

Furthermore, because few studies have focused on the secular trends of HPV subtypes in China, we also analyzed the trends from 2014 to 2018 in our hospital. Differences were determined for subtypes HPV39, 51, 52 and 58, and HPV39 and 51 were significantly increased in recent years. Based on the results of our study and others, there was variation of HPV prevalence by geographic regions, age distribution and time. Therefore, the effectiveness of vaccination for reduction of disease burden may differ by population. Currently, the 9v HPV vaccine has been widely used clinically and it has recently been shown to prevent HPV infection among subjects with differing baseline characteristics [33]. However, vaccines including more HPV genotypes should be developed for the Beijing area, such as HPV52, 16, 58, 39 and 51, and others.

TCT is the most advanced cytological examination technique for cervical cancer globally, and its detection rate for cervical cells is near to 100% [34]. TCT represents significant progress in collecting cytological samples and preparing slides, and the technique has been certified by the US FDA and is recommended by the College of American Pathologists to replace the traditional cervical smear. In our study, a total of 1746 (8.22%) women were detected as TCT positive. The highest TCT-positive rate was seen in the 40–50-year age group, and the lowest in the 30–40-year age group. In addition, the reason for the higher TCT-positive rate in women aged < 30 years may be associated with the higher frequency of HPV infection in this group. However, there is little age-specific variation in TCT-positive rate. Additionally, among four cytological subtypes, HSILs were detected significantly more with increasing age. Among single- and multiple-type HPV infection, the TCT-positive rate was significantly higher in the latter. Among the four types of cytological lesions, the HPV infection rate was 16.75, 66.08, 63.99 and 85.32% for NILM, ASC, LSIL and HSIL, respectively. Our results indicated that HR-HPV infection was associated with the development of cervical lesions and the HSIL group had the highest HPV infection rate. Therefore, it was an important hint for patients with abnormal cytological results to be subjected HPV detection.

The present study included 21,239 patients between 2014 and 2018, which identified the most common HPV subtypes among women. However, there were some limitations to this study. First, this study was only carried out in a single center in the Beijing area, and more samples and multicenter data need to be detected. Second, we only detected 13 HR-HPV subtypes (16, 18, 31, 33, 35, 39, 45, 51, 52, 56, 58, 59 and 68), and the other HPV subtypes such as 66 and 82 should be included in further study. Third, although we analyzed the relationship between cervical cytology and HPV genotypes, the histology was not available for correlation with HPV genotypes.

## Conclusions

In summary, this retrospective study demonstrated that the three most prevalent HR-HPVs were in descending order of HPV52, 16 and 58. The secular trends from 2014 to 2018 in our data reflected that HPV39 and 51 were increasing during recent years and that HSILs increased significantly with age. Our data provide valuable information for HPV-based screening and prevention strategies for women in Beijing area. Because of differences in HPV subtypes between China and western countries, next-generation HPV vaccines including HPV52, 16, 58, 39 and 51 should be considered in the near future.

## Data Availability

The datasets used and/or analysed during the current study are available from the corresponding author on reasonable request.

## Abbreviations

ASCs: Atypical squamous cells
HPV: Human papillomavirus
HR-HPV: High-risk HPV
HSILs: High-grade squamous intraepithelial lesions
LSILs: Low-grade squamous intraepithelial lesions
TCT: ThinPrep cytological test
